# Gold(I) Weak Binding Sites: Histidine Residues of Serum Albumin

**DOI:** 10.1155/MBD.1994.519

**Published:** 1994

**Authors:** J. Xiao, C. Frank Shaw III

**Affiliations:** 1 Department of Chemistry University of Wisconsin-Milwaukee Milwaukee WI 53201-0413 USA


					GOLD(I) WEAK BINDING SITES:
HISTIDINE RESIDUES OF SERUM ALBUMIN
J. Xiao and C. Frank Shaw I!1
Department of Chemistry, University of Wisconsin-Milwaukee, Milwaukee, Wl-53201-0413, USA
Et3PAuCI, an analogue of the antiarthritic drug auranofin [Et3PAuSATg triethylphosphine-(2,3,4,6-
tetra-O-acetyl-l-thio-8-D-glucopyranosato-S)gold(I)] binds to weak binding sites of serum albumin
in vitro. Up to 17 equivalents of Et3PAu+ react with bovine serum albumin (BSA). These weak
binding sites are characterized by 31p NMR shifts of 23-29 ppm and may be the seventeen histidine
residues.
In order to investigate these sites, a 1H NMR study was performed. With a spin echo pulse
sequence (.=30ms), most of the C2H resonances (7.5-8.5 ppm) of the histidine imidazole rings of
BSA were resolved. Their assignments as the C2H of the histidines were confirmed by a 1H NMR
pH titration. Upon titrating BSA with Et3PAuCI, shifting and broadening of these C2H resonances
occurred. Et3PAu+ bound preferentially to the more hydrophobic histidine residues characterized
by shorter T2 relaxation times. All the resonances were eventually undetectable. The same
phenomena (broadening and shifting of the C2H resonance) were observed in model studies in
which L-histidine and N-acetyI-L-histidine were titrated with Et3PAuCI.
519

				

